# Practical Medicine Series

**Published:** 1912-08

**Authors:** 


### Books and Magazines.

Practical Medicine Series.—Ten Volumes on the Year's Progress in Medicine and Surgery. Year-Book Publishers, Chicago. Series of 1912.

This series is published primarily for the general practitioner, and is arranged so that a single volume may be purchased by one interested in any special subject. The set of ten is sold for $10. Single volume, $1.25 to $2.00. Each volume is complete for the year prior to its publication. Volumes 1 (Surgery) and 2 (General Medicine) have been announced. We now have Volume 3, The Eye, Ear, Nose and Throat; a beauty. It is edited by Wood, Andrews and Head, all recognized authorities, and is beautifully illustrated. Price $1.25 by mail.

